# Neural frequency distributions may generate a new phase transition in models for synchronization

**DOI:** 10.1186/1471-2202-15-S1-P155

**Published:** 2014-07-21

**Authors:** Marcelo HR Tragtenberg, Caio L Tiedt, Mauricio Girardi-Schappo

**Affiliations:** 1 Department of Physics, Federal University of Santa Catarina, 88040-900, Florianópolis, SC, Brazil

## 

Neural synchronization is a phenomenon related to information transmission between brain areas, cognitive functions, perceptual and motor skills and memory [1,2], and also connected to mental illnesses like epilepsy, isolated seizures, Alzheimer´s disease, Parkinson´s disease, autism and schizophrenia [3]. This work is related to the critical state of the brain [4]: the order of the synchronization transition. We study this subject computationally using two models: Kuramoto’s [5] and the KTz model [6].

The Kuramoto model is a paradigm in the study of synchronization. The order parameter for the synchronization exhibits a phase transition from a non-synchronized to a synchronized phase that can be first-order for a uniform distribution of the individual frequencies of the coupled oscillators, or second-order in the case of Lorentzian or Gaussian distributions [8]. The KTz is a formal neuron map-based model that can be coupled by map-based chemical synapses and also can exhibit synchronization phase transition under certain circumstances.

We study the transition between a uniform to a unimodal frequency distribution in the Kuramoto. This change in the frequency distribution is made through a particular kind of discretization of the Gaussian and Lorentzian distributions: we create distributions that are podia with *2m*+*1* steps. We study the cases from the uniform distribution (*m* = *0*) to a continuous unimodal distribution (*m*→ *∞*). There is an abrupt transition in the behavior of the model as a function of *m:* for m>1 the transition is always second order. We sum a noise term in the oscillator frequencies and in the interaction between oscillators, separately. For each of the two cases, the phase transition in the order parameter for the uniform distribution frequency seems to change the order from first to second.

We also have preliminary results for synchronization in the KTz model, studying an order parameter analog to that for the Kuramoto model [10]. The synchronization transition seems to be second-order as a function of the connection strength, as it should be to for a critical brain.
